# Connaissances, attitudes et pratiques relatives à la cataracte et au glaucome dans la population de Conakry en Guinée

**DOI:** 10.11604/pamj.2022.42.9.30311

**Published:** 2022-05-06

**Authors:** Maxime Dantouma Sovogui, Pierre Louis Lamah, Christophe Zoumanigui, Tamba Elie Tolno, Kokou Vonor

**Affiliations:** 1 Clinique Ophtalmologique Bartimée, Conakry, Guinée Conakry,; 2 Université de Conakry, Faculté des Sciences et Techniques de la Santé, Conakry, Guinée Conakry; 3 Centre Hospitalier Régional de Kara, Kara, Togo,

**Keywords:** Connaissances, attitudes et pratiques (CAP), Bartimée, Conakry, Guinée, Knowledge, attitudes and practices (KAP), Bartimée, Conakry, Guinea

## Abstract

**Introduction:**

le but de ce travail est d´évaluer les connaissances, attitudes et pratiques (CAP) relatives à la cataracte et au glaucome dans la population de Conakry Guinée.

**Méthodes:**

nous avions mené une étude prospective de type descriptif et analytique de trois mois à la clinique Bartimée. Etaient inclus, les patients qui ont accepté de répondre à notre questionnaire et ayant un âge≥18 ans. Les questions CAP sur cataracte et glaucome leur étaient posées. Les niveaux de connaissance ont été corrélés à l´âge, au sexe et au niveau d´instruction.

**Résultats:**

au total, 1000 personnes ont participé à l´étude. Le sex-ratio était de 1,10 et l´âge moyen 42,41 ans ± 21,74. Les Hommes de métiers étaient plus représentés 2,80%; 45,10% était analphabète; 47,50% avaient un très bon niveau de connaissance; 59,10% des patients savaient que le traitement de la cataracte est chirurgical. Pour le glaucome: 55,80% des patients pensaient que le traitement est chirurgical. Face à la cataracte et au glaucome 51,90% avaient déclaré partir à l´hôpital, 38,80% iront consulter un guérisseur traditionnel et 9,30% feront une automédication. Il existait un lien significatif entre l´âge, le sexe et le niveau d´instruction avec les connaissances sur la cataracte et le glaucome

**Conclusion:** à Conakry, les CAP sur la cataracte et le glaucome sont moins satisfaisantes. Des stratégies de sensibilisation devraient être entreprises pour améliorer ses résultats.

## Abstract (en)

**Introduction:**

the purpose of this study was to evaluate the knowledge, attitudes and practices (KAP) related to cataract and glaucoma in the population of Conakry Guinea.

**Methods:**

we conducted a prospective descriptive and analytical study at the Bartimée Clinic over a period of three months. All patients who agreed to answer our questionnaire and having an age ≥18 years were included. KAP questions related to cataract and glaucoma were asked. Knowledge levels were correlated with age, gender and education.

**Results:**

a total of 1000 people participated in the study. Sex-ratio was 1.10 and the mean age was 42.41 years ± 21.74. Male occupations were found to be more represented 21.80%; illiterate persons accounted for 45.10%, those with a very good level of knowledge 47.50%, patients knowing that the treatment of cataract was surgical 59.10%. For glaucoma: 55.80% of patients thought that the treatment was surgical. In the case of cataract and glaucoma 51.90% said they would go to hospital, 38,80% would go to a traditional healer and 9,30% practiced self-medication. There was a significant link between age, gender and education level and knowledge about cataract and glaucoma.

**Conclusion:**

in Conakry, Guinea, KAP related to cataract and glaucoma are less than satisfactory. Awareness-raising strategies should be undertaken to improve results.

## Introduction

La cataracte se définit comme une opacification partielle ou totale du cristallin responsable d´une diminution de l´acuité visuelle [1]. Elle constitue la première cause de cécité dans le monde et touche 18 millions de personnes; elle est la cause de 50% des cas de cécité en Afrique et en Asie [2,3]. Son seul traitement de nos jours demeure chirurgical. La connaissance de cette affection par la population est essentielle dans la prévention de la cécité [4]. Le glaucome est une neuropathie optique progressive qui se manifeste par des anomalies de la papille optique et des altérations subséquentes du champ visuel [5,6]. C´est l´une des principales causes de cécité irréversible dans le monde [7-9]. Les données des enquêtes basées sur la population indiquent que le glaucome est la deuxième cause de cécité [8]. En 2013, le nombre de personnes âgées de 40 à 80 ans atteintes de glaucome dans le monde était estimé à 64,3 millions, passant à 76,0 millions en 2020 et 111,8 millions en 2040 [7]. Bien que le glaucome soit irréversible, il existe des options de traitement par lesquelles la progression de la maladie peut être retardée. Ces méthodes comprennent le traitement médical, chirurgical et physique.

Un diagnostic rapide et un respect strict du traitement sont nécessaires pour la réussite de la prise en charge du glaucome. Les connaissances et les attitudes des patients vis-à-vis du glaucome sont des facteurs importants dans sa prise en charge [10]. En effet, les connaissances des populations sur les pathologies sont en général insuffisantes et les attitudes, parfois erronées, voire néfastes. Une enquête sur les CAP est un instrument participatif pour la promotion de la santé [11]. Les connaissances et les attitudes individuelles des maladies oculaires sont des facteurs importants dans le dépistage, le diagnostic, la prévention et la compliance au traitement. Il est donc recommandé de renforcer les stratégies de prévention, y compris le développement des ressources humaines. L´une des conditions fondamentales requises pour une bonne méthode de prévention est l´évaluation du niveau de connaissance de la maladie et du niveau de conscientisation [4]. Au Togo en 2011, dans une interview réalisée auprès d´un échantillon opportuniste de 802 personnes âgées de 20 ans et plus, la connaissance du glaucome est notée chez 296 enquêtés soit 36,9% dont 39 étaient glaucomateux soit 13,2% [12]. Au Mali Bamako, Aboubakar H *et al*. dans une étude transversale descriptive pendant une période de 10 jours au cours d´une campagne de soins ophtalmologiques, 83,7% des patients avaient des connaissances justes sur la cataracte. Vingt-quatre soit 4,3% des patients savaient que le traitement est chirurgical et 242 soit 43,8% pensaient que le traitement est traditionnel [4]. Vue l´attitude très variante des individus vis-à-vis de la cataracte et du glaucome, de même que la perception qu´ils en ont selon les pays et même à l´intérieur d´un même pays; aussi l´une des conditions de base pour juger une stratégie de prévention étant l´évaluation du niveau de connaissance de la population, de son appréhension du problème et de sa démarche, cette étude se propose d´évaluer les CAP des populations concernant la cataracte et le glaucome à Conakry en Guinée.

## Méthodes

**Conception de l'étude**: il s´agit d´une étude prospective de type descriptif et analytique d´une durée de trois mois allant du 1^er^ septembre au 31 décembre 2020 au cours des consultations ophtalmologiques. Elle s´est déroulée dans la clinique ophtalmologique Bartimée qui est un établissement hospitalier de second degré et spécialisé en ophtalmologie. Elle est située au quartier Nongo, secteur I, commune de Ratoma, Conakry.

**Participants à l'étude**: un total de 1000 patients reçus en consultations ophtalmologiques à la clinique Bartimée a participé à l´étude. Etaient inclus dans cette étude, les patients qui ont accepté de répondre à notre questionnaire et ayant un âge supérieur ou égal à 18 ans. N´ont pas été inclus de cette étude, tous les patients chez qui le consentement libre et éclairé n´a pas été obtenu.

**Echantillonnage**: nous avions procédé à un échantillonnage de préférence d´une taille de 1000 échantillons tous venus en consultation à la clinique ophtalmologique Bartimée.

**Instrument de collecte de données**: les questions par rapport à la cataracte et au glaucome ont été posées sous forme d´interview en langue locale ou en français afin d´évaluer les niveaux de CAP de nos patients à travers un questionnaire établi à cet effet qui comprenait trois (3) parties: La première partie concernait l´identification des répondants (Age, sexe, profession, provenance, niveau d´instruction). La deuxième partie concernait huit (8) questions sur les connaissances des répondants sur la cataracte (quatre questions) et le glaucome (quatre questions) à savoir définition de la cataracte et du glaucome? La cataracte et le glaucome peuvent-ils rendre aveugle? La cataracte et le glaucome sont-ils curables? Quels sont les moyens de traitement? La troisième partie comprenait les attitudes et pratiques des participants face à la cataracte et au glaucome, à la question de savoir: que faites-vous face à la cataracte et au glaucome? Nous avions opté des scores d´évaluation des niveaux de connaissances de nos patients sur la cataracte et le glaucome de la façon suivante: Chaque bonne réponse a été notée 1 point, les mauvaises réponses aux questions et les réponses « ne sait pas » ont reçu un score de zéro. Le score maximum à atteindre était de 10 points. Après calcul des scores, nous procédions au classement de celles-ci selon les intervalles suivants: les scores de 1 à 3 qui correspondent au niveau de connaissance bas; les scores de 4 à 7 correspondent au niveau de connaissance bon et les scores de 8 à 10 au niveau de connaissance très bon. Ce qui nous a permis de classer nos patients selon leurs niveaux de connaissance. La cataracte était décrite comme une tache blanche dans l´œil, associée à une baisse de la vue et le glaucome comme l´augmentation de tension dans les yeux. Les paramètres étudiés étaient l´âge, le sexe, la profession, la provenance, le niveau d´instruction, les CAP concernant la cataracte et le glaucome.

**Analyse des données**: les données ont été traitées et analysées par le logiciel Epi-info version 7.4.0, saisi à l´aide des logiciels Word et Excel du pack office 2013. Le khi carré de Person a été utilisé pour la comparaison des niveaux de connaissance de nos patients sur la cataracte et le glaucome avec les variables sociodémographiques à savoir le sexe, l´âge et le niveau d´instruction et Une valeur p inférieure à 0,05 a été considérée comme statistiquement significative dans toutes les analyses. Le logiciel Zotero dans sa version 5.0.96.2 a été utilisé pour les références bibliographiques.

**Aspects éthiques et règlementaires**: la confidentialité et l´anonymat des personnes enquêtées ont été respectés conformément aux principes de l´éthique et de la déontologie médicale.

## Résultats

**Figure 1:**
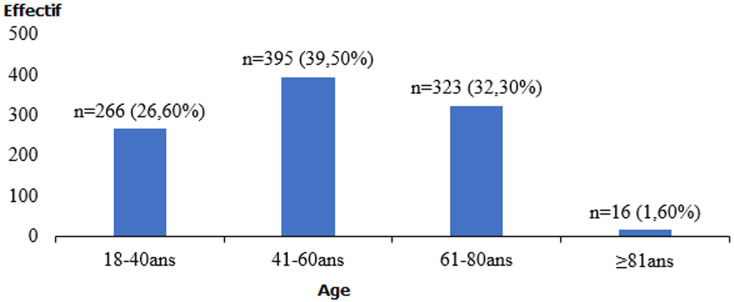
répartition des répondants selon l´âge (n=1000)

**Caractéristiques sociodémographiques des répondants**: au total, 1000 personnes ont participé à notre étude dont 524 hommes soit 52,40% et 476 femmes soit 47,60% avec un sex-ratio de 1,10. L´âge moyen était de 42,41 ans ± 21,74 avec des extrêmes de 18 ans et 93 ans. La tranche d´âge la plus représentée était celle de 41 à 60 ans soit 39,50% suivis de 61-80 ans, 323 cas soit 32,30%, de 18-40 ans 266 cas soit 26,60%, et ceux ≥81 ans 16 cas soit 1,6% (Figure 1). Les Hommes de métiers et les élèves/étudiants étaient les plus représentés soit respectivement 21,8% et 19% suivis des Fonctionnaires 187 cas soit 18,70%, des ménagères 184 cas soit 18,40%, des marchands 162 cas soit 16,20%, des sans-emplois 38 cas soit 3,8%, des agents de sécurités 18 cas soit 1,8% et des hommes religieux 3 cas soit 0,3%. Concernant le niveau d´instruction, 451 patients soit 45,10% étaient analphabètes, 364 cas soit 36,40% avaient un niveau d´instruction supérieur et 185 cas soit 18,50% un niveau d´instruction secondaire.

**Connaissances des répondants sur la cataracte et le glaucome**: la grande majorité des personnes enquêtées, 698 cas soit 69,80% savait définir la cataracte comme une tache blanche dans l´œil, suivi de 189 cas soit 18,90% qui disaient ne pas savoir définir la cataracte, 69 cas soit 6,9% la considérèrent comme une maladie mystique. Cependant 44 patients soit 4,4% disaient que la cataracte est une opacification du cristallin. Aussi 750 d´entre eux soit 75% connaissaient que la cataracte peut rendre aveugle et 757 soit 75,70% croyaient à sa curabilité parmi lesquels, 591 soit 59,10% disaient que le traitement est chirurgical, 228 soit 22,80% des personnes pensaient qu´il est médicamenteux, par contre 92 personnes soit 9,20% pensaient que le traitement est traditionnel et pour 89 cas soit 8,90%, elle se soigne par le port des verres de lunettes. Par rapport au glaucome, 416 patients soit 41,60% disaient simplement que c´est une maladie des yeux alors que 242 patients soit 24,20% ne connaissaient pas cette maladie et 107 patients soit 10,70% pensaient que c´est un mauvais sort. Il était défini comme une augmentation de tension dans les yeux par 94 patients soit 9,40%; 50 patients soit 5% le définissaient comme une maladie mystique et 50 cas 5% comme une complication d´autres maladies. Il était considéré comme une tache blanche dans l´œil pour 24 patients soit 2,40% et comme des gros yeux pour 17 patients soit 1,70%. Il existe des liens statistiquement significatifs entre les niveaux de connaissance de nos patients sur la cataracte et le glaucome avec les variables sociodémographiques à savoir le sexe, l´âge et le niveau d´instruction (Tableau 1). Les hommes, les sujets âgés de plus de 60 ans et ceux ayant un niveau d´instruction supérieur avaient une meilleure connaissance sur la cataracte et le glaucome.

**Tableau 1:** répartition des répondants selon leurs niveaux de connaissance en fonction du sexe, de l´âge et du niveau d´instruction (n=1000)

Sexe	Niveau de connaissance			Total	P
	Bas	Bon	Très bon		
**Masculin**	122	73	329	524	0,000
**Féminin**	180	150	146	476	
**Tranche d´âge (année)**					0,000
18-45	120	66	80	266	
41-60	85	130	180	395	
61-80	90	23	210	323	
≥ 81	7	4	5	16	
**Niveau d´instruction**					0,000
**Analphabète**	200	146	105	451	
**Secondaire**	32	12	141	185	
**Supérieur**	70	65	229	364	

**Attitudes et pratiques des répondants face à la cataracte et au glaucome**: en ce qui concerne les attitudes et pratiques vis-à-vis de la cataracte et du glaucome, 519 patients soit 51,90% avaient déclaré partir à l´hôpital; 388 patients soit 38,80% iront consulter un guérisseur traditionnel et 93 patients soit 9,30% feront une automédication (Tableau 2).

**Tableau 2:** répartition des répondants selon l´attitude et pratique devant la cataracte et le glaucome (n=1000)

Attitude/pratique	Effectif	Pourcentage
Aller à l´hôpital	519	51,90
Aller chez le tradipraticien	388	38,80
Automédication	93	9,30

## Discussion

Parmi les 1000 patients interrogés, il y avait une prédominance masculine avec un sex-ratio de 1,10. Contrairement à Aghedo AV *et al*. [10] qui rapportaient un sex-ratio de 0,73. L´âge moyen était de 42,41 ans ± 21,74 avec des extrêmes de 18 ans et 93 ans. Cependant la tranche d´âge la plus représentée était celle de 41 à 60 ans soit 39,50%. Notre résultat est proche de celui de Aboubakar H *et al*. [4] qui ont retrouvé que plus de 20% de leur population d´étude avait plus de 60 ans; d´autres auteurs comme Tham YC *et al*. [7] et Delgado MF *et al*. [9] confirment ce résultat. Cela pourrait s´expliquer par le fait que dans cette tranche d´âge, la fréquence de la cataracte liée à l´âge est très élevée [13] et que l´incidence du glaucome augmente avec l´âge [14]. Les Hommes de métiers et les élèves/étudiants étaient les plus représentés soit respectivement 21,8% et 19%. Ceci est une indication du faible revenu économique de cette population. Par ailleurs, les revenus économiques sont corrélés avec la cécité et la fréquence élevée des maladies oculaires [4]. Ceci, parce que l´accès aux soins ophtalmologiques en général et à la chirurgie de la cataracte et du glaucome en particulier est conditionné par les moyens pécuniaires. Près de la moitié de nos patients provenaient de l´intérieur du pays avec 356 patients soit 35,60% et une majorité analphabète avec 451 patients soit 45,10%. Ceci s´observe souvent en milieu rural dans les pays en voie de développement. Thapa SS *et al*. [15] en 2011 dans une étude menée en zone rurale au Népal avaient aussi obtenu une fréquence élevée de sujets analphabètes soit 53,25%.

Dans notre étude, la grande majorité soit 69,80% savait définir la cataracte. Notre résultat est comparable à celui de Vonor K *et al*. [13] au Togo qui ont mené leur étude en milieu rural. En plus, près de 75% des patients de cette population reconnait que la cataracte peut rendre aveugle. Aboubakar H *et al*. [4] ont rapporté un résultat semblable. Plus de la moitié de cette population 591 soit 59,10% disaient que le traitement de la cataracte est chirurgical suivi de 228 soit 22,8% des personnes qui pensaient qu´il est médicamenteux, par contre 92 personnes soit 9,2% pensaient que le traitement est traditionnel. C´est le contraire dans l´étude d´Aboubakar H *et al*. [4] qui ont rapporté que c´est une infime minorité de leur population qui savait que le traitement est chirurgical et que la majorité pensait qu´il n´y a aucun traitement et 43,80% croyait au traitement traditionnel. Par rapport au glaucome, 416 patients soit 41,60% disaient simplement que c´est une maladie des yeux et Il était défini par 94 patients soit 9,40% comme une augmentation de la tension dans les yeux. Notre résultat corrobore avec celui de Ayena K.D *et al*. [12] au Bénin qui ont rapporté que la connaissance du glaucome était notée chez 296 de leurs enquêtés soit 36,90%. Selon 558 patients soit 55,80% le traitement du glaucome est chirurgical, il est médical pour 335 patients soit 33,5% et traditionnel pour 45 patients soit 4,5%. Ayena K.D *et al*. [12] rapportent des proportions similaires. Il existe des liens statistiquement significatifs entre les niveaux de connaissance sur la cataracte et le glaucome avec les variables sociodémographiques à savoir le sexe, l´âge et le niveau d´instruction soit p < 0,05 dans chacun des cas. En effet, nous avions retrouvé que les hommes, les sujets âgés de plus de 60 ans et ceux ayant un niveau d´instruction supérieur avaient une meilleure connaissance sur la cataracte et le glaucome. Ces résultats corroborent ceux d´Aboubakar H *et al*. [4], qui avaient rapporté une meilleure connaissance chez les hommes, les instruits et les sujets plus âgés. Thapa SS *et al*. [15] trouvaient une corrélation positive entre le niveau d´instruction et celui de la connaissance sur la cataracte. En ce qui concerne les attitudes vis-à-vis de la cataracte et du glaucome, 519 patients soit 51,9% avaient déclaré partir à l´hôpital; 388 patients soit 38,8% iront consulter un guérisseur traditionnel et 93 patients soit 9,3% feront une automédication. Ces résultats sont contraires à ceux d'Aboubakar H *et al*. [4] au Mali qui trouvaient que 51,10% de la population avait déclaré ne rien faire devant une cataracte et 43,8% serait prêt à consulter un guérisseur traditionnel et seulement 5,10% aurait la volonté de se rendre dans un hôpital pour traiter une cataracte.

## Conclusion

A Conakry, les CAP sur la cataracte et le glaucome sont moins satisfaisantes. Dans la majorité des cas les hommes, les sujets âgés de plus de 60 ans et ceux ayant un niveau d´instruction supérieur ont une bonne connaissance sur la cataracte et le glaucome. Cependant cette connaissance demeure superficielle car les attitudes et pratiques restent dans l´ensemble inadéquates. Des actions vigoureuses de campagnes de sensibilisation et de soins oculaires devraient être entreprises pour améliorer ses résultats.

### Etat des connaissances sur le sujet

- La cataracte et le glaucome sont des pathologies ophtalmologiques les plus fréquentes en Guinée;
- La cataracte et le glaucome sont respectivement la première et la troisième cause de chirurgie à la Clinique ophtalmologique Bartimée;
- Le recourt à notre service n´est pas toujours en première intention.

### Contribution de notre étude à la connaissance

- Cette étude a évalué la connaissance, les attitudes et pratiques de la population, liées à la cataracte et au glaucome;
- Cette étude a démontré que les CAP sur la cataracte et le glaucome sont moins satisfaisantes dans notre pays;
- Cette étude a prouvé que les connaissances de la population demeurent superficielles car les attitudes et pratiques restent dans l´ensemble inadéquates.
